# The Antenatal Origins of Postpartum Distress: A Retrospective Longitudinal Analysis of Depression and Anxiety Trajectories

**DOI:** 10.3390/medsci14010102

**Published:** 2026-02-19

**Authors:** Larisa-Mihaela Holbanel, Adina Turcu-Stiolica, Sebastian Constantin Toma, Mihail-Cristian Pirlog, Victor Gheorman

**Affiliations:** 1 Doctoral School, University of Medicine and Pharmacy of Craiova, 200349 Craiova, Romania; dr.larisamihaela@gmail.com; 2 Biostatistics Department, Faculty of Pharmacy, University of Medicine and Pharmacy of Craiova, 200349 Craiova, Romania; 3 Health Economics and Outcomes Research Department, “Iuliu Haţieganu” University of Medicine and Pharmacy Cluj-Napoca, Victor Babes Street No. 8, 400012 Cluj-Napoca, Romania; 4 General Surgery Department, Dr. Constantin Andreoiu, Ploiesti County Emergency Hospital, 100097 Ploiesti, Romania; tosecon@hotmail.com; 5 Medical Sociology Department, Faculty of Medicine, University of Medicine and Pharmacy of Craiova, 200349 Craiova, Romania; mihail.pirlog@umfcv.ro; 6 Psychiatry Department, Faculty of Medicine, University of Medicine and Pharmacy of Craiova, 200349 Craiova, Romania; victor.gheorman@umfcv.ro

**Keywords:** pregnancy, depression, Edinburgh Postnatal Depression Scale, Generalized Anxiety Disorder Questionnaire, perinatal mental health

## Abstract

**Background/Objectives**: While postpartum depression is widely screened, the predictive value of antenatal symptoms remains underutilized. This study aimed to retrospectively analyze the longitudinal trajectory of depressive and anxiety symptoms from mid-pregnancy to the late postpartum period to identify critical windows for intervention and assess the impact of mental health service utilization. **Methods**: A retrospective longitudinal cohort study was conducted on 125 pregnant women monitored at the Emergency County Clinical Hospital of Craiova, Romania. Depression was assessed using the Edinburgh Postnatal Depression Scale (EPDS) and Patient Health Questionnaire-9 (PHQ-9), while anxiety was evaluated using the Generalized Anxiety Disorder-7 (GAD-7) scale. Data were collected at four intervals: 20–24 weeks (T1), 32–36 weeks (T2), 6 weeks postpartum (T3), and 12 weeks postpartum (T4). **Results**: The highest burden of depressive symptoms occurred in the antenatal period (mean EPDS: 15.6 ± 9.41) rather than postpartum. Antenatal depression scores were strongly correlated with postpartum scores (rho = 0.98, *p* < 0.001), indicating a stable continuum of distress, though this high correlation may also reflect measurement inertia. Anxiety scores demonstrated a “plateau” effect during pregnancy (mean GAD-7 ≈ 8.0) before declining postpartum. A stratified analysis revealed a “treatment paradox”: women receiving mental health services had higher baseline morbidity and a slower rate of recovery compared to those who did not, remaining symptomatic at 12 weeks (mean EPDS: 14.2 vs. 11.0, *p* = 0.049). **Conclusions**: Perinatal distress in this cohort was primarily an antenatal phenomenon that persisted in the postpartum period. The “antenatal peak” suggests the hypothesis that screening should commence in the second trimester. Current interventions appear to stabilize but not fully resolve symptoms in high-risk women, suggesting a need for more intensive management strategies.

## 1. Introduction

Perinatal depression is among the most common yet underdiagnosed complications of pregnancy and the postpartum period, with substantial consequences for maternal, fetal and neonatal health [1,2,3]. Its impact extends beyond maternal well-being, influencing clinical obstetrical outcomes, mother–infant interaction and long-term child development [4,5,6]. Emerging evidence suggests that the pathophysiology of perinatal mood disorders extends into the intrauterine environment, influencing both gestational duration and neonatal vitality [5]. Furthermore, the disruption of the postpartum bonding process serves as a mechanism through which maternal depressive symptoms may exert long-term effects on both maternal caregiving behaviors and infant socio-emotional development [4].

Pregnancy represents a critical window of vulnerability for the onset or exacerbation of mood and anxiety disorders [7,8]. The convergence of neurohormonal dysregulation, psychosocial adversity and obstetric risk factors creates a risk profile that is further moderated by an individual’s psychiatric history [3,9].

Validated self-report instruments, such as the Edinburgh Postnatal Depression Scale (EPDS), Patient Health Questionnaire-9 (PHQ-9) and Generalized Anxiety Disorder-7 (GAD-7), are widely used to assess depressive and anxiety symptoms in perinatal populations [10,11,12]. While the EPDS is specifically designed for use during pregnancy and the postpartum period, PHQ-9 and GAD-7 provide complementary information on depressive severity and anxiety symptoms [13]. Despite their extensive validation, few longitudinal studies have applied these instruments concurrently across multiple antenatal and postpartum time points to examine symptom trajectories and their predictive relationships [14,15,16].

Despite the global focus on perinatal mental health, there is a paucity of longitudinal data from Eastern European settings where screening protocols are not yet universally mandated. This study addresses this gap by providing “real-world” retrospective data from a Romanian clinical cohort. The primary objective was to analyze the longitudinal trajectory of depressive and anxiety symptoms from mid-pregnancy (20–24 weeks) to the late postpartum period (12 weeks). The secondary objective was to evaluate the impact of mental health service utilization on symptom recovery, with the objective of informing evidence-based timing of perinatal mental health screening.

## 2. Materials and Methods

We conducted a retrospective longitudinal cohort study at the Emergency County Clinical Hospital of Craiova, Romania, analyzing data collected between 20 July 2024 and 30 June 2025. The study protocol was approved by the Ethics Committee of the University of Medicine and Pharmacy of Craiova (Approval no. 196/18.07.2024). All data were anonymized for analysis, and written informed consent for the use of medical data for research purposes was obtained from all participants at the time of their initial clinical assessment.

### 2.1. Study Participants

This study was designed as a retrospective longitudinal cohort study. Data were collected retrospectively from existing medical records and routinely administered mental health screening questionnaires. No prospective recruitment, randomization, or intervention was performed as part of this study. We analyzed the medical records of pregnant women monitored at the Emergency County Hospital of Craiova, Romania, between 20 July 2024 and 20 September 2025. We extracted the de-identified data from existing clinical files, where mental health screening was performed as part of a perinatal care protocol.

In the present study, mental health services refer to standard clinical care pathways available within the health care system, including screening, referral and counseling when indicated. No structured psychological intervention, psychotherapy program, or experimental mental health treatment was implemented or evaluated as part of this study. Referrals to mental health professionals followed routine clinical instructions and were independent of study objectives.

We included women aged >18 years, with singleton pregnancies, who attended all four scheduled monitoring visits (20–24 weeks gestation, 32–36 weeks gestation, 6 weeks postpartum, and 12 weeks postpartum) and who had complete mental health screening data for these time points. We excluded patients with incomplete medical records, those who withdrew from care before the 12-week postpartum mark, and those with a pre-existing diagnosis of psychotic disorders or cognitive impairment that precluded valid questionnaire completion.

Demographic and clinical variables were extracted from the patients’ charts, including age, place of residence (urban/rural), education level, marital status, parity, gestational age, mode of birth, and history of complications or comorbidities.

Information regarding mental health service utilization was extracted as a binary variable (service received/no service received). Due to the retrospective nature of the data, granular details regarding the specific type (e.g., psychotherapy vs. pharmacotherapy), intensity, and duration of the interventions were not consistently available. This limitation prevents a dose–response analysis of treatment efficacy.

### 2.2. Assessment of Depression and Anxiety

The late pregnancy and postnatal questionnaires included the validated Romanian version of the EPDS, a 10-item instrument commonly used in prenatal care for depression screening [17]. The EPDS is the most used depression screening tool in postnatal care. It has a scale of 0 to 30, and individuals who score ≥ 13 are considered likely to be suffering from perinatal depression [10].

Anxiety was measured with the Romanian GAD-7 at four time points: in the second trimester, in the third trimester, 6 weeks after birth, and 12 weeks after birth. This 7-item instrument measures the frequency of anxiety symptoms over the preceding two weeks, with total scores ranging from 0 to 21 [12,18]. A cut-off of 10 was recommended for diagnosis of generalized anxiety disorder with a sensitivity of 89% and specificity of 82% [19].

Depression severity in early pregnancy was assessed by the Romanian PHQ-9, utilizing a 4-point Likert scale (0–27 points) [20]. Total scores of 5, 10, 15, and 20 represent cut-points for mild, moderate, moderately severe and severe depression, respectively.

This study followed the Strengthening the Reporting of Observational Studies in Epidemiology (STROBE) reporting guidelines for cohort studies [21].

### 2.3. Statistical Analyses

Continuous variables (e.g., age, gestational age) were reported as mean ± standard deviation (SD) or median (interquartile range, IQR) depending on distribution. Categorical variables were presented as frequencies and percentages. The Shapiro–Wilk test was applied to check the distribution of depression and anxiety scores. As the data followed a non-normal distribution (*p* < 0.001), non-parametric tests were employed. Changes in EPDS, PHQ-9, and GAD-7 scores across the time points were analyzed using the Friedman test for repeated measures. Significant results were followed by Wilcoxon signed-rank tests with Bonferroni correction for pairwise comparisons. A complete case analysis was performed, excluding participants with missing data points across the four intervals. We utilized the non-parametric Friedman test for repeated measures due to the non-normal distribution of the data. We acknowledge that, unlike mixed-effects modeling, this approach does not fully account for time-varying covariates or within-subject correlation structures, representing a methodological limitation of the analysis.

The relationships between sociodemographic factors and mental health scores were evaluated using Spearman’s rank correlation coefficient. Differences in symptom trajectories between patients who received mental health services and those who did not were assessed using the Mann–Whitney U test. A *p*-value of <0.05 was considered statistically significant for all analyses. Data analysis was performed using Python (version 3.6, Pandas/SciPy libraries). Given the retrospective design and the fixed size of the clinical cohort (N = 125), a priori sample size calculation was not performed. However, a post hoc power analysis was performed using G*Power software (version 3.1.9.7) to confirm that the sample size provided high power to detect strong longitudinal correlations (Spearman’s ρ > 0.5) at α = 0.05.

## 3. Results

### 3.1. Descriptive Analysis

The study cohort comprised 125 women with a mean age of 28.9 ± 6.42 years, as shown in Table 1. The majority of participants resided in urban environments (*n* = 82, 65.6%) and were married (*n* = 95, 76%). Regarding educational background, a significant proportion of the sample had completed secondary or higher education, with 37.6% holding a university degree and 36.8% having completed high school. Obstetric characteristics revealed a balanced distribution between primiparous (51.2%) and multiparous (48.8%) women, with a mean gestational age of 24.2 ± 9.96 weeks. Spontaneous delivery was the predominant mode of birth (60.8%), while 28% of the cohort reported complications in previous pregnancies. Regarding clinical history, 27.2% of participants documented a personal history of depression, with an identical prevalence observed for family history of the disease. While the majority of patients (63.2%) presented no somatic comorbidities, 15.2% were diagnosed with hypertension, 14.4% were diagnosed with diabetes, and 7.2% presented with both conditions.

The means ± standard deviation and medians (interquartile range) of maternal depression and anxiety scores before and after births are shown in Table 2. High baseline morbidity was observed during pregnancy. The mean EPDS score in the antepartum period was 15.6 ± 9.41, with a median of 16 (IQR: 7–24), indicating substantial depressive symptom burden in the cohort. Specific symptom analysis revealed a diverse clinical presentation: anxiety was the most prevalent predominant symptom (24%), followed closely by sadness (20.8%) and lack of interest (19.2%). Notably, no participants reported active suicidal thoughts during the initial assessment.

The PHQ-9 and GAD-7 scores remained stable throughout the second half of pregnancy. Mean scores of depression measured by PHQ-9 were 12.4 ± 7.95 at T2 and 12.6 ± 7.77 at T3, reflecting a plateau of moderate depressive symptoms. Mean scores of anxiety measured by GAD-7 were 8.0 ± 4.98 at T2 and 7.99 ± 4.88 at T3, consistent with mild-to-moderate anxiety that did not fluctuate significantly as the pregnancy progressed.

Following delivery (mean assessment day: 22.8 ± 12.4), a general trend of symptom reduction was observed across all scales, though mean scores remained clinically significant. The mean EPDS score decreased to 14.0 ± 9.45. Similarly, PHQ-9 scores dropped to 11.3 ± 7.81, and GAD-7 scores declined to 6.82 ± 4.73. Further improvement was noted by the 12-week mark. The mean EPDS score fell to 12.6 ± 9.14, the PHQ-9 to 10.0 ± 7.45, and the GAD-7 to 6.16 ± 5.43. Despite this downward trend, the mean EPDS score at 12 weeks (12.6) remained near the standard screening cut-off for probable depression.

### 3.2. Assessment of Depressive Symptoms Across the Perinatal Period

A longitudinal assessment of depressive symptoms was conducted across three distinct time points, antepartum (baseline), 6 weeks postpartum, and 12 weeks postpartum, using the EPDS. Descriptive statistics revealed a progressive decline in mean depression scores over time. The highest burden of depressive symptoms was observed in the antepartum period, with a mean EPDS score of 15.55 ± 9.41. At the first postpartum assessment (6 weeks), the mean score decreased to 14.02 ± 9.45 and further declined to 12.58 ± 9.14 at 12 weeks postpartum. Due to the non-normal distribution of the data (Shapiro–Wilk test, *p* < 0.001), a non-parametric Friedman test was performed to evaluate differences across the three time points. The test indicated a statistically significant difference in EPDS scores over time (χ^2^(2) = 116.53, *p* < 0.001).

**Figure 1:**
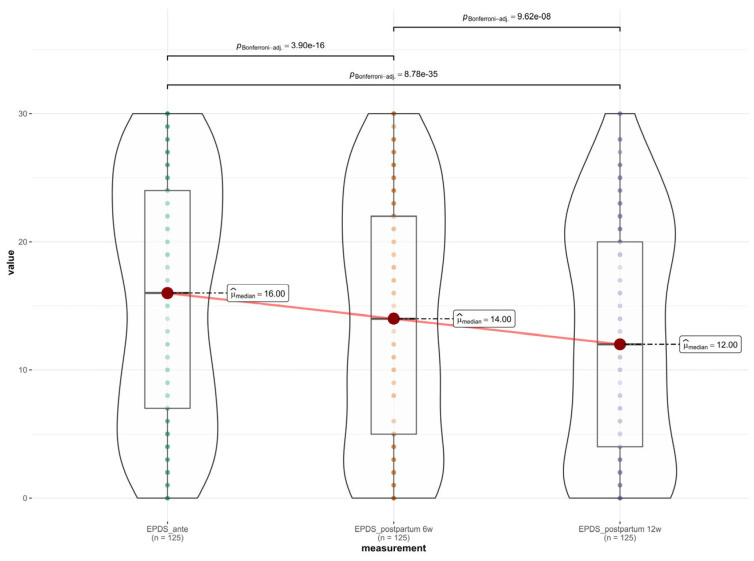
Depressive symptoms across the perinatal period.

Post hoc pairwise comparisons using the Wilcoxon signed-rank test with Bonferroni correction confirmed that the reduction in scores was significant at each stage: antepartum vs. 6 weeks postpartum: significant decrease (*p* < 0.001); 6 weeks vs. 12 weeks postpartum: significant decrease (*p* < 0.001); and antepartum vs. 12 weeks postpartum: significant decrease (*p* < 0.001), as shown in Figure 1.

### 3.3. Assessment of Depressive Symptom Severity Across the Perinatal Period

A longitudinal analysis of depressive symptom severity was performed using the Patient Health Questionnaire-9 (PHQ-9) across four distinct perinatal intervals: T1 (20–24 weeks gestation), T2 (32–36 weeks gestation), T3 (6 weeks postpartum), and T4 (12 weeks postpartum).

**Figure 2:**
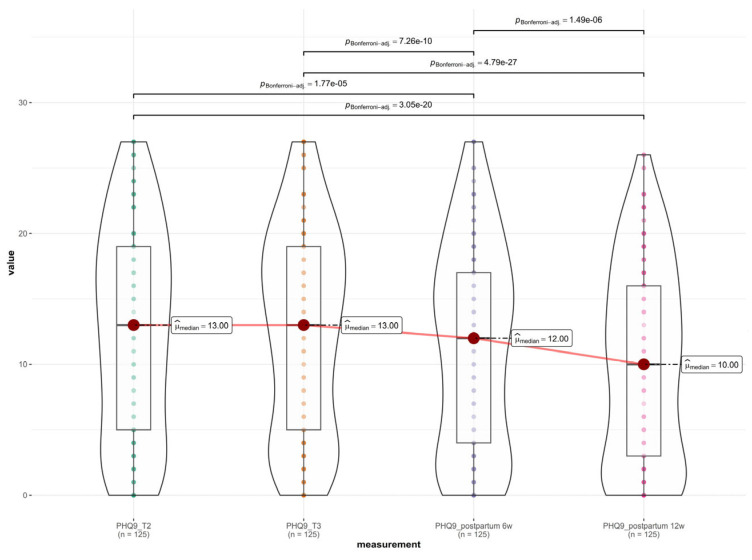
Depressive symptom severity across the perinatal period.

Descriptive statistics indicated a specific trajectory of symptom severity. Mean PHQ-9 scores remained stable and elevated during the antenatal period, rising slightly from 12.44 ± 7.95 at T1 to a peak of 12.59 ± 7.77 at T2. Following delivery, a progressive decline was observed, with scores dropping to 11.31 ± 7.81 at 6 weeks postpartum and further to 10.05 ± 7.45 at 12 weeks postpartum, as shown in Figure 2.

The non-parametric Friedman test confirmed a statistically significant difference in depression scores across the four time points (χ^2^(3) = 117.35, *p* < 0.001).

Post hoc pairwise comparisons using the Wilcoxon signed-rank test yielded some specific dynamics regarding pregnancy progression (T1 vs. T2), effect of birth (T2 vs. T3), postpartum recovery (T3 vs. T4), and overall trajectory (T1 vs. T4). There was no statistically significant difference between the second and third trimesters (*p* = 0.32). This indicates that depressive symptoms remained uniformly high throughout the second half of pregnancy. A highly significant decrease was observed between the third trimester and 6 weeks postpartum (*p* < 0.001). A further significant decrease occurred between 6 weeks and 12 weeks postpartum (*p* < 0.001). The final score at 12 weeks was significantly lower than the baseline antenatal score (*p* < 0.001).

### 3.4. Assessment of Anxiety Symptoms Across the Perinatal Period

**Figure 3:**
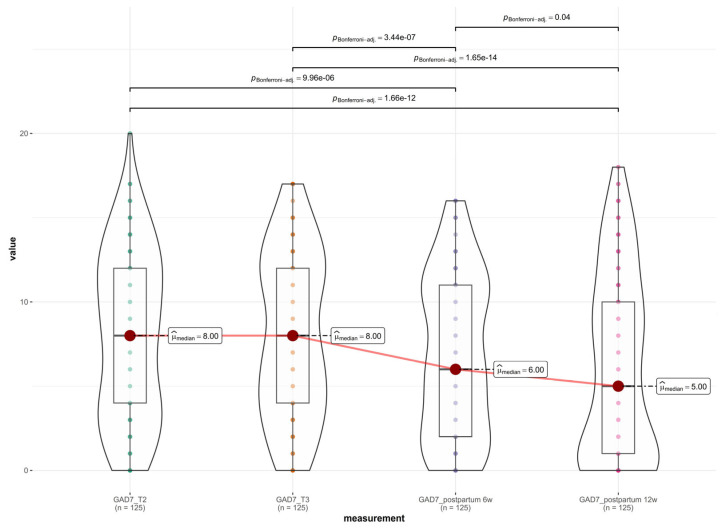
Anxiety symptoms across the perinatal period.

A longitudinal assessment of anxiety symptoms was conducted using the Generalized Anxiety Disorder-7 (GAD-7) scale across four key perinatal intervals, T1 (20–24 weeks gestation), T2 (32–36 weeks gestation), T3 (6 weeks postpartum), and T4 (12 weeks postpartum), as shown in Figure 3. Descriptive statistics revealed a trajectory remarkably similar to that of the depression scores. Anxiety levels remained stable and elevated throughout the second half of pregnancy, with mean scores of 8.00 ± 4.98 at T1 and 7.99 ± 4.88 at T2. Following delivery, a statistically significant decline was observed, with mean scores dropping to 6.82 ± 4.73 at 6 weeks postpartum and further to 6.16 ± 5.43 at 12 weeks postpartum. The non-parametric Friedman test indicated a statistically significant difference in anxiety severity across the four time points (χ^2^(3) = 75.57, *p* < 0.001). Post hoc pairwise comparisons using the Wilcoxon signed-rank test elucidated some specific dynamics: antenatal stability (T1 vs. T2), effect of birth (T2 vs. T3), postpartum recovery (T3 vs. T4), and overall trajectory (T1 vs. T4). There was no significant difference in anxiety levels between the second and third trimesters (*p* = 0.90), indicating a plateau of symptoms during pregnancy. A highly significant reduction in anxiety occurred between the third trimester and 6 weeks postpartum (*p* < 0.001). A further significant decline was observed between 6 weeks and 12 weeks postpartum (*p* < 0.001). Anxiety levels at 12 weeks postpartum were significantly lower than the antenatal baseline (*p* < 0.001).

### 3.5. Correlations with Depressive and Anxiety Symptoms and Characteristics of the Patients

The Spearman correlation heatmap reveals two distinct clusters of variables: a high-correlation cluster involving all mental health scores (depression and anxiety symptoms across all timelines) and a low-correlation cluster involving sociodemographic and obstetric history variables.

**Figure 4:**
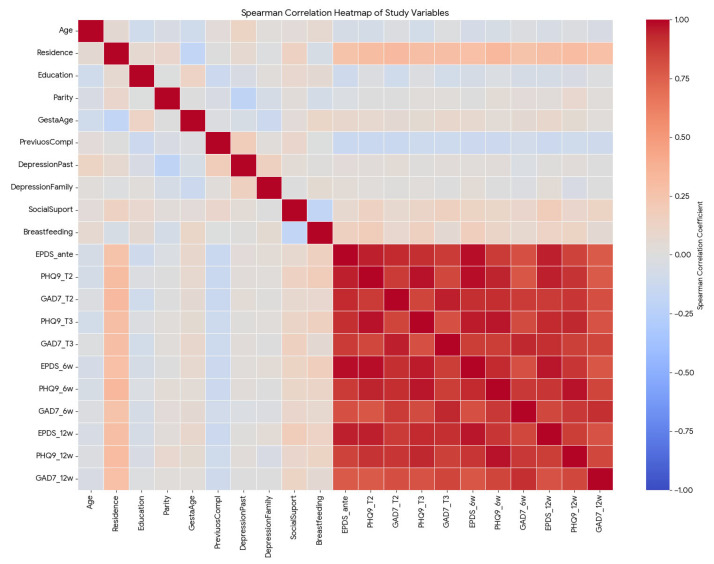
Anxiety symptoms across the perinatal period. Red (positive correlation) indicates that as one variable increases, the other also increases. Darker red represents a stronger positive association (approaching rho = +1.0). The intense red cluster in the bottom-right illustrates the strong continuity between antenatal and postpartum mental health scores. Blue (negative correlation) indicates an inverse relationship (as one variable increases, the other decreases). Darker blue represents a stronger negative association (rho = −1.0). White/light colors indicate weak or negligible correlations (rho = 0). The visual distinction clearly separates the high-correlation cluster of mental health symptoms from the low-correlation cluster of demographic factors.

Figure 4 is a strong visual of the “red block” of high correlations between antepartum and postpartum scores. There is a near-perfect positive correlation between antenatal depression scores and postpartum scores. For instance, EPDS (antepartum) and EPDS (6 weeks postpartum) have a correlation coefficient of rho = 0.98, *p* < 0.0001. This indicates that the depression level during pregnancy is almost the sole predictor of depression after birth in this cohort.

Depression (EPDS/PHQ-9) and anxiety (GAD-7) scores are strongly correlated at every time point (rho > 0.80, *p* < 0.0001). Patients with high depression scores almost invariably present with high anxiety.

Place of residence shows a moderate positive correlation (rho > 0.28, *p* < 0.01) with depression scores, suggesting that patients in urban settings (coded as 1) tended to have higher depression scores than those in rural settings (coded as 0).

Variables such as parity, gestational age, and previous complications showed negligible correlations (rho < 0.15, *p* < 0.05) with the mental health outcomes, indicating these biological factors were not primary drivers of depression in this group.

A weak positive correlation (rho = 0.18, *p* = 0.049) was observed, implying either a slight paradoxical association where patients reporting “Yes” to mental health services had marginally higher scores or that the support variable did not function as a protective buffer in this specific dataset for depressive symptoms at 12 weeks postpartum.

Post hoc power analysis was conducted to evaluate the statistical robustness of the results, and it yielded a statistical power of 98% at a significance level of 0.05. These results confirm that the sample size was sufficient to validate the primary study hypotheses despite the limitations of a single-center cohort.

### 3.6. Longitudinal Trajectory of Depression and Anxiety Symptom Scores by Mental Health Service Utilization

**Figure 5:**
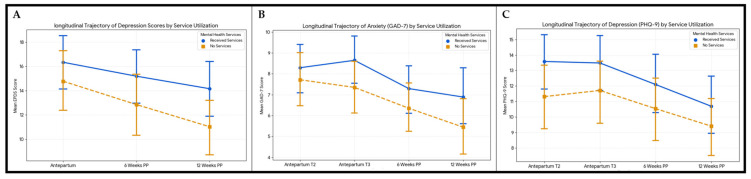
Longitudinal trajectory of depression symptoms and anxiety in peripartum by mental health service utilization. (**A**) EPDS scores; (**B**) GAD-7 scores; (**C**) PHQ-9 scores.

A stratified longitudinal analysis was performed to compare depression score trajectories (EPDS) between patients who received mental health services (Group A, *n* = 62) and those who did not (Group B, *n* = 63). At the antenatal baseline, the group receiving mental health services presented with higher mean depression scores compared to the non-service group, although this difference did not reach statistical significance (16.34 ± 8.9 vs. 14.78 ± 9.9, *p* = 0.39). This trend suggests a “selection by indication,” where patients with higher symptom burdens were more likely to be identified for support. A general trend of symptom reduction was observed for both groups: at 6 weeks postpartum, Group B significantly improved compared to Group A (*p* = 0.011), but at 12 weeks postpartum the trend was similar (*p* = 0.093). At 12 weeks postpartum, Group A remained at a mean EPDS ± SD of 14.2 ± 8.81 (still above the screening cut-off of 13), whereas Group B had dropped to 11.03 ± 9.26 (below the cut-off), as shown in Figure 5A.

Mean scores of anxiety (GAD-7) were 8.3 ± 4.65 for Group A and 7.71 ± 5.31 for Group B at T2 and 8.65 ± 4.62 for Group A and 7.35 ± 5.09 for Group B at T3, consistent with mild-to-moderate anxiety that did not fluctuate significantly as the pregnancy progressed. At 6 weeks postpartum, the non-service group showed a slightly greater reduction in anxiety compared to the service group, but this was not significant (*p* = 0.28). The reduction was similar from 6 to 12 weeks postpartum for both groups (*p* = 0.34), as shown in Figure 5B.

Similar to the EPDS findings, the group receiving services had higher baseline depression scores of PHQ-9 (13.58 ± 7.42) compared to the non-service group (11.32 ± 8.35), though this difference was not statistically significant (*p* = 0.11) in the second trimester. Almost the same scores were observed at T3, reflecting a plateau of moderate depressive symptoms. Both groups improved at 6 weeks postpartum, though the difference among them was not statistically significant (*p* = 0.28). From 6 to 12 weeks postpartum, both groups continued to improve at similar rates (*p* = 0.34), as shown in Figure 5C.

## 4. Discussion

This longitudinal cohort study provides a granular assessment of the trajectory of perinatal mental health in 125 women, tracking symptoms of depression and anxiety from the second trimester of pregnancy through 12 weeks postpartum. Our findings challenge several traditional paradigms regarding the onset and course of perinatal mood disorders, highlighting the critical importance of antenatal screening and the limitations of current standard-of-care interventions.

The most significant finding of this study is that the highest burden of depressive symptoms occurred during the antenatal period (mean EPDS: 15.6) rather than in the postpartum period. This contradicts the historical emphasis on “postpartum depression” as a distinct, new-onset phenomenon triggered by childbirth. Our data aligns with recent findings by Mladenovic et al. (2024), who similarly reported a higher prevalence of depressive symptoms during pregnancy compared to the postpartum period (13.5% vs. 4.8%) in a tertiary hospital setting [22]. The near-perfect correlation found between antenatal and postpartum scores (rho = 0.98) suggests a deterministic relationship or a “continuum of distress.” However, we must also consider the possibility of measurement inertia or construct overlap, where repeated administration of the same self-report instruments over a short period may result in response redundancy: the mental health status of the mother at 20–24 weeks gestation is the single strongest predictor of her status at 12 weeks postpartum. This consistency across cohorts supports the “continuum of distress” hypothesis, suggesting that for the majority of women, postpartum depression is actually the continuation of untreated antenatal depression. Consequently, screening protocols that commence only after delivery likely miss the critical window for preventative intervention.

Our analysis of GAD-7 scores revealed a distinct “anxiety plateau” during the second half of pregnancy, with no significant fluctuation between 20 and 36 weeks (*p* = 0.90). This stability argues against the theory that anxiety is solely driven by immediate fear of childbirth (tokophobia), suggesting instead a generalized, trait-like burden. This finding is reinforced by the systematic review from Rondung et al. (2023), which emphasizes the complex etiology of antenatal anxiety and its high comorbidity with depression, arguing that single-disorder screening is insufficient [23]. With 24% of patients reporting anxiety as their predominant symptom, our data confirms the mixed affective state of perinatal distress. Similarly, Solomonov et al. (2025) found in a large urban cohort (N = 27,393) that 23.2% of screened women reported clinically meaningful symptoms, with a high overlap between depression and anxiety domains [24].

A striking observation was that patients receiving mental health services demonstrated a slower rate of recovery compared to those who did not. This finding should be interpreted through the lens of “confounding by indication” rather than treatment inefficacy. The service group presented with higher baseline severity, suggesting that clinicians correctly identified and referred the most symptomatic women. The persistence of pathological scores at 12 weeks likely reflects a severity bias, where the treated cohort possesses a more complex, treatment-resistant phenotype (e.g., chronic history, unmeasured psychosocial stressors). This indicates that while standard interventions are stabilizing these high-risk patients, they may be insufficient to achieve full remission. This contrasts with the findings of Solomonov et al. (2025), who reported that women receiving mental health services had faster reductions in depression severity over time compared to untreated women (*p* < 0.001) [24]. This divergence likely reflects a “severity bias” in our smaller cohort: services were utilized by the most symptomatic, treatment-resistant patients, whereas Solomonov’s large-scale mandatory screening likely identified and treated milder cases that responded more readily to intervention [24]. This discrepancy highlights a critical gap in our local standard of care: while we are identifying high-risk women, the current interventions appear to stabilize rather than cure them.

The persistence of pathological scores at 12 weeks postpartum (mean EPDS: 12.6) is clinically concerning. Zhang et al. (2025) recently demonstrated that maternal mood disorders persisting into the late postpartum period are associated with significantly larger reductions in infant health-related quality of life compared to transient early symptoms [25]. This underscores that the “natural decline” in scores we observed (15.6 → 12.6) is statistically significant but clinically insufficient. The window of risk extends well beyond the standard 6-week check-up, necessitating prolonged surveillance [26].

Our correlation analysis identified urban residence as a stronger correlate of depression than biological factors such as age, parity, or obstetric complications. This highlights the role of environmental stressors—potentially including social isolation, economic pressure, and fragmented support networks—typical of urban living. Interestingly, “Social Support” showed a weak positive correlation with symptoms, likely reflecting that support was mobilized in response to visible distress, rather than acting as a pre-emptive buffer. This finding aligns with the “mobilization hypothesis,” where higher symptoms elicit more help, yet this help is often reactive rather than preventative.

Several limitations must be acknowledged. First, this study was conducted at a single center, potentially limiting generalizability to broader rural or culturally distinct populations compared to multi-site studies like those reviewed by Rondung et al. [23]. Second, the sample size (*n* = 125) limits the statistical power for robust subgroup analyses. Third, the classification of “mental health services” was binary, preventing a detailed analysis of the efficacy of specific modalities (e.g., CBT vs. medication). Because this is a retrospective analysis, we could not prove causation (e.g., that treatment caused slower recovery), but we could observe the strong associations. The 12-week follow-up period may be insufficient to capture late-onset postpartum depression or long-term recovery. Finally, while the attrition rate was low, the absence of a pre-pregnancy baseline prevents us from determining if the high antenatal scores represent new-onset pregnancy depression or chronic pre-existing dysthymia.

## 5. Conclusions

This study demonstrates that in this cohort, the burden of depressive and anxiety symptoms peaked in the antenatal period and showed strong continuity into the postpartum phase. These findings support the clinical utility of initiating screening in the second trimester. The observed persistence of symptoms in women receiving services highlights the complexity of treating high-risk perinatal populations and suggests that current standard-of-care interventions may need to be intensified or extended. Future prospective, multi-center studies are needed to validate these trajectories and evaluate specific stepped-care interventions.

## Figures and Tables

**Table 1:** Sociodemographic baseline characteristics of the included women.

Characteristics	Total N = 125
Age, years	28.9 ± 6.42
	29 (24–33)
Place of residence	
Urban	82 (65.6%)
Rural	43 (34.4%)
Education level	
Primary school	11 (8.8%)
Middle school	21 (16.8%)
High school	46 (36.8%)
University	47 (37.6%)
Marital status	
Married	95 (76%)
Unmarried	14 (11.2%)
Divorced	16 (12.8%)
Parity	
Primiparous	64 (51.2%)
Multiparous	61 (48.8%)
Gestational age, weeks	24.2 ± 9.96
	23 (16–33)
Previous complications, yes	35 (28%)
Birth type	
Spontaneous	76 (60.8%)
Cesarean delivery	49 (39.2%)
Depression in the past, yes	34 (27.2%)
Depression in the family, yes	34 (27.2%)
Symptoms	
Anxiety	30 (24%)
Insomnia	22 (17.6%)
Irritability	23 (18.4%)
Lack of interest	24 (19.2%)
Sadness	26 (20.8%)
Comorbidities	
None	79 (63.2%)
Diabetes	18 (14.4%)
Hypertension	19 (15.2%)
Diabetes + hypertension	9 (7.2%)

Continuous data are presented as mean ± standard deviation, median (interquartile range). Categorical data are presented as frequency (percentages).

**Table 2:** Depression and anxiety scores across the perinatal period.

Characteristics		Total N = 125			
	Antepartum 24 Weeks	Antepartum 36 Weeks	Postpartum 6 Weeks	Postpartum 12 Weeks	*p*-Value (Friedman Test)
EPDS	15.6 ± 9.41 16 (7–24)		14 ± 9.45 14 (5–22)	12.6 ± 9.14 12 (4–20)	<0.001
PHQ9	12.4 ± 7.95 13 (5–19)	12.6 ± 7.77 13 (5–19)	11.3 ± 7.81 12 (4–17)	10 ± 7.45 10 (3–16)	<0.001
GAD7	8 ± 4.98 8 (4–12)	7.99 ± 4.88 8 (4–12)	6.82 ± 4.73 6 (2–11)	6.16 ± 5.43 5 (1–10)	<0.001

EPDS, Edinburgh Postnatal Depression Scale; PHQ9, Patient Health Questionnaire; GAD7, Generalized Anxiety Disorder Questionnaire.

## Data Availability

The original data presented in the study are openly available in Zenodo (version v1.0.) at DOI 10.5281/zenodo.18194733.

## Abbreviations

The following abbreviations are used in this manuscript:

EPDS	Edinburgh Postnatal Depression Scale
PHQ-9	Patient Health Questionnaire-9
GAD-7	Generalized Anxiety Disorder-7
